# Twenty-four hour metabolic rate measurements utilized as a reference to evaluate several prediction equations for calculating energy requirements in healthy infants

**DOI:** 10.1186/1475-2891-10-14

**Published:** 2011-02-02

**Authors:** Russell Rising

**Affiliations:** 1 D & S Consulting Services Inc., 1 Horizon Rd, #1407, Fort Lee, NJ 07024 USA

## Abstract

**Background:**

To date, only short-duration metabolic rate measurements of less than four hours have been used to evaluate prediction equations for calculating energy requirements in healthy infants. Therefore, the objective of this analysis was to utilize direct 24-hour metabolic rate measurements from a prior study to evaluate the accuracy of several currently used prediction equations for calculating energy expenditure (EE) in healthy infants.

**Methods:**

Data from 24-hour EE, resting (RMR) and sleeping (SMR) metabolic rates obtained from 10 healthy infants, served as a reference to evaluate 11 length-weight (LWT) and weight (WT) based prediction equations. Six prediction equations have been previously derived from 50 short-term EE measurements in the Enhanced Metabolic Testing Activity Chamber (EMTAC) for assessing 24-hour EE, (EMTACEE-LWT and EMTACEE-WT), RMR (EMTACRMR-LWT and EMTACRMR-WT) and SMR (EMTACSMR-LWT and EMTACSMR-WT). The last five additional prediction equations for calculating RMR consisted of the World Health Organization (WHO), the Schofield (SCH-LWT and SCH-WT) and the Oxford (OXFORD-LWT and OXFORD-WT). Paired t-tests and the Bland & Altman limit analysis were both applied to evaluate the performance of each equation in comparison to the reference data.

**Results:**

24-hour EE, RMR and SMR calculated with the EMTACEE-WT, EMTACRMR-WT and both the EMTACSMR-LWT and EMTACSMR-WT prediction equations were similar, p = NS, to that obtained from the reference measurements. However, RMR calculated using the WHO, SCH-LWT, SCH-WT, OXFORD-LWT and OXFORD-WT prediction equations were not comparable to the direct 24-hour metabolic measurements (p < 0.05) obtained in the 10 reference infants. Moreover, the EMTACEE-LWT and EMTACRMR-LWT were also not similar (p < 0.05) to direct 24-hour metabolic measurements.

**Conclusions:**

Weight based prediction equations, derived from short-duration EE measurements in the EMTAC, were accurate for calculating EE, RMR and SMR in healthy infants.

## Background

There are many prediction equations currently in use to calculate energy requirements in healthy infants [1-4]. These are popular among many health care practitioners due to their ease of use. For many of these equations only length and weight of the infants need to be measured prior to calculations. Some of the most commonly utilized equations for infants include those from the World Health Organization [1], Schofield [2] and Oxford [3]. However, some of these prediction equations [1,2] were based on limited data obtained over 80 years ago utilizing non-standardized techniques [3,5]. Derivation of all of these prediction equations [1-4] were based on short-term metabolic measurements. For example, only 30-45 minute measurements of resting metabolic rates in individuals were utilized for the derivation of the World Health Organization [1], Schofield [2] and Oxford equations [3]. In an attempt to improve the accuracy of calculating energy requirements for infants, new prediction equations derived and published from our laboratory [4] were based on 50 four-hour morning metabolic measurements in the Enhanced Metabolic Testing Activity Chamber (EMTAC).

The EMTAC was applied in the first ever direct 24-hour measurement of energy expenditure, resting and sleeping metabolic rates in both healthy [6] and in those infants recovering from malnutrition [7]. The aim of this analysis was to use data from previously published direct 24-hour metabolic measurements in healthy infants [6] as a reference to evaluate 11 prediction equations for calculating 24-hour energy expenditure, resting and sleeping metabolic rates [1-4].

## Methods

### Subjects

Data from 10 healthy full term formula fed (Carnation^® ^Good Start with iron) infants that were part of a previous study of 24-hour metabolic rate [6] were utilized to evaluate several prediction equations for calculating 24-hour energy expenditure (EE), resting (RMR) and sleeping (SMR) metabolic rates. The ethnic mix of the healthy infants consisted of nine Hispanics and one Afro-American. All anthropometric, growth performance [8] and 24-hour metabolic data for the 10 healthy reference infants used in this evaluation are shown in Table 1. The 24-hour metabolic data utilized in this analysis was from a published study previously approved by the Institutional Review Board of Miami Children's Hospital, Miami Florida, USA [6].

**Table 1:** Anthropometric, growth performance and metabolic data (Mean ± SD) for the healthy reference infants [6]

Parameter	N = 10
Males/Females	7/3

Age (months)	5.0 ± 0.8

Length (cm)	68.8 ± 2.8

BMI (kg/m^2^)	15.5 ± 1.5

Length-for-age percentile	81.5 ± 14.3

Weight-for-age percentile	65.0 ± 21.0

Weight-for-length percentile	27.5 ± 23.1

24-h Energy expenditure (kcal/kg/d)	78.7 ± 8.4

Resting metabolic rate (kcal/kg/d)	66.0 ± 3.5

Sleeping metabolic rate (kcal/kg/d)	65.0 ± 3.4

### Direct measurement of 24-hour metabolic rate

Each of the 10 reference infants spent 24-hours in the Enhanced Metabolic Testing Activity Chamber (EMTAC) for measurement of energy expenditure, resting and sleeping metabolic rate as previously published from our laboratory [6]. However, a brief description of the methodology is provided. Prior to each metabolic measurement the EMTAC was calibrated with standard gases with a known concentration of oxygen and carbon dioxide. Furthermore, parents were given instruction on how to interact with their infants and were given time to practice using the hand access ports prior to metabolic testing. Each infant was placed in the EMTAC for 24-hours from 9:30 AM till 9:29 AM the following day for continuous measurements of energy expenditure (EE; kcal/min), physical activity (PA; oscillations in weight/min/kg body weight) and the respiratory quotient (RQ:VCO_2_/VO_2_). Any supplies such as diapers, formula, baby food or toys were placed in the EMTAC in hanging bags before the start of the test. Parents continued to formula feed their infants at their discretion during metabolic testing.

Energy expenditure (kcal/min) was continuously calculated during metabolic testing according to the method of Jequier [9] and summarized every five minutes as described previously [10].

There were no restrictions in regards to room lighting, feeding or interaction of the infant or with any of the activities of the family during the entire testing procedure. One of the four investigators (RR, MC, DD and SV) acted as observers on rotating eight hour shifts and recorded all infant activities such as infant feedings, periods of observed sleep and amount of parental interaction during the entire 24-hour testing period.

At the conclusion of each metabolic test, all metabolic data were corrected for parental interaction, prior to the calculation of resting (RMR; kcal/kg/d) and sleeping metabolic rates (SMR; kcal/kg/d). This involved eliminating any five-minute EE summary period where parents interacted with their infants. Thereafter, RMR was calculated by regressing EE on PA, multiplying the resulting y-intercept by 1440 (minutes in 24-hours). Twenty-four hour SMR was calculated by retaining all EE periods between 11:30 PM and 5:30 AM where the index of PA was less than or equal to 1.5 and the infant was observed to be asleep. The mean of these EE periods was multiplied by 1440. This is similar to the methodology used for calculating SMR in adults [11]. All metabolic results were expressed as kcal/kg/d.

### Calculations

Twenty-four hour energy expenditure, RMR and SMR were estimated from 11 previously published prediction equations, utilizing the length and weight of the 10 reference infants (Table 1), in order to allow for comparisons between indirect calculations and the direct 24-hour measurement of the energy expenditure components. More specific, six of these equations (Table 2) were from a previous study in our laboratory [4] where 50 short-term metabolic measurements in infants of four-hours in the EMTAC were used in their derivation. These consisted of two equations for 24-hour EE based on both length and weight (EMTACEE-LWT) and weight (EMTACEE-WT), two equations for RMR based on both length and weight (EMTACRMR-LWT) and weight (EMTACRMR-WT) and finally, two equations for SMR based on both length and weight (EMTACSMR-LWT) and weight (EMTACSMR-WT). Additionally, five other prediction equations (Table 2) [1-3] such as the World Health Organization (WHO), Schofield length-weight based (SCH-LWT), Schofield weight-based (SCH-WT), Oxford length-weight based (OXFORD-LWT) and Oxford weight-based (OXFORD-WT) were also compared to direct 24-hour resting (basal) metabolic rates [6] derived from the EMTAC.

**Table 2:** Current prediction equations for infants 0-3 years of age used in our analysis

Source	Sex	Prediction equation (kcal/kg/d)
EMTACEE-WT	Males or females	(98.1 × WT ) - 121.7

EMTACEE-LWT	Males or females	(10.7 × L) + (73.3 × WT) - 635.1

EMTACRMR-WT	Males or females	(84.5 × WT) - 117.3

EMTACRMR-LWT	Males or females	(10.1 × L) + (61.0 × WT) - 605.1

EMTACSMR-WT	Males or females	(73.3 × WT) - 72.6

EMTACSMR-LWT	Males or females	(7.6 × L) + (55.6 × WT) - 440.7

WHO	Males	(60.9 × WT) - 54.0
	Females	(61.0 × WT) - 51.0

SCH-WT	Males	(59.5 × WT) - 30.3
	Females	(58.3 × WT) - 31.1

SCH-LWT	Males	(0.2 × WT) + (1517.4 × (L/100)) - 617.6
	Females	(16.3 × WT) + (1023.2 × (L/100)) - 413.5

OXFORD-WT	Males	(61.0 × WT) - 33.7
	Females	(58.9 × WT) - 23.1

OXFORD-LWT	Males	(28.2 × WT) + (859.0 × (L/100)) - 371.0
	Females	(30.4 × WT) + (703 × (L/100)) -287.0

### Statistical Analysis

All data were analyzed with SPSS software (v13; Chicago, IL) and expressed as Mean ± Standard Deviation (SD) at the 5% level of probability (p < 0.05). The 24-hour data from the reference infants was normally distributed as previously published [6] therefore parametric statistics was utilized for the data analysis. Moreover, the sample size was appropriate for this analysis based on the results from previous short [4,10,12] and long term [6,7] metabolic studies with the EMTAC instrument.

Two different statistical analyses were conducted utilizing the anthropometric and sex data from the 10 healthy reference infants. First, paired t-tests were performed to compare the metabolic results calculated using the EMTACEE-LWT, EMTACEE-WT, EMTACRMR-LWT, EMTACRMR-WT, EMTACSMR-LWT, EMTACSMR-WT, WHO, SCH-LWT, SCH-WT, OXFORD-LWT and OXFORD-WT prediction equations [1-4] to the respective 24-hour measured metabolic parameter from the EMTAC [6]. Second, the Bland and Altman limit analysis [13] was also performed to determine agreement between the reference metabolic parameter determined for 24-hours in the EMTAC to that calculated with each of the prediction equations being evaluated [13]. This involves taking the average between each reference and respective calculated value ((reference + calculated value/2) for each infant and comparing the mean result across all 10 infants to the mean differences (reference - calculated value) between that particular reference and respective calculated value. Mean differences with a close proximity to zero (no differences between the reference and calculated values) suggests good agreement while mean differences close to two standard deviations from zero (large differences between reference and calculated values) suggest poor agreement [13].

All metabolic data were expressed as kcal/kg/d where necessary by dividing the results from each of the prediction equations by the infant's body weight in kg.

## Results

Energy expenditure (EE), resting (RMR) and sleeping metabolic rates (SMR) of the 10 healthy reference infants used for this evaluation, as directly measured for 24-hours in the EMTAC, are shown in Table 1. Calculated results from the EMTACEE-WT, EMTACRMR-WT, EMTACSMR-WT and EMTACSMR-LWT prediction equations were in agreement (p = NS) with that obtained for the reference infants (Table 3). The percentage difference from the 10 healthy reference infants that had direct 24-hour metabolic rate measurements was less than 5% when utilizing these prediction equations for calculating 24-hour EE, RMR and SMR (Table 3). This was further verified by the fact that the mean differences calculated utilizing the Bland and Altman limit analysis method had a close proximity to zero or were close to the mean value (Table 3).

**Table 3:** Statistical analysis for prediction equations that are in agreement with direct 24-hour metabolic measurements

Prediction	Value	**Difference**^**1**^	P	**Agreement**^**2**^
equation	(Mean ± SD)	(%)	(Paired T-test)	(Bland & Altman ± 2SD)
EMTACEE-WT	81.3 ± 1.8	4.5 ± 12.0	0.42	2.6 ± 19.6

EMTACRMR-WT	65.0 ± 3.9	4.0 ± 7.9	0.16	2.4 ± 9.8

EMTACSMR-WT	63.3 ± 1.1	-2.2 ± 7.4	0.23	-1.7 ± 9.6

EMTACSMR-LWT	66.7 ± 2.4	3.0 ± 8.2	0.29	1.8 ± 10.0

^1 ^= ((calculated value from the corresponding prediction equation-reference metabolic parameter/reference metabolic parameter)*100)

^2 ^= Proximity to zero calculated as the difference between the reference metabolic parameter and the calculated value from the corresponding prediction equation (± 2 standard deviations) [13]

Calculated results from the EMTACEE-LWT, EMTACRMR-LWT, WHO, SCH-LWT, SCH-WT, OXFORD-LWT, OXFORD-WT prediction equations were not in agreement (p < 0.05) with that obtained for the reference infants (Table 4). The percentage difference from the 10 healthy reference infants that had direct 24-hour metabolic rate measurements was greater than 11% when utilizing these prediction equations for calculating 24-hour EE and RMR (Table 4). This was further verified by the fact that average differences calculated utilizing the Bland and Altman limit analysis method were close to, or over two standard deviations from the mean (Table 4).

**Table 4:** Statistical analysis for prediction equations that are not in agreement with direct 24-hour metabolic measurements

Prediction	Value	**Difference**^**1**^	P	**Agreement**^**2**^
Equation	(Mean ± SD)	(%)	(Paired T-test)	(Bland & Altman ± 2SD)
EMTACEE-LWT	86.6 ± 3.3	11.2 ± 12.7	<0.01	7.9 ± 19.2

EMTACRMR-LWT	73.3 ± 3.2	11.4 ± 9.3	<0.01	7.3 ± 11.2

WHO	53.6 ± 0.8	-18.6 ± 5.5	<0.01	-12.4 ± 8.2

SCH-WT	54.9 ± 0.8	-16.6 ± 5.1	<0.01	-11.1 ± 7.6

SCH-LWT	57.9 ± 5.6	-12.2 ± 9.0	<0.01	-8.1 ± 11.8

OXFORD-WT	56.2 ± 0.6	-14.7 ± 5.2	<0.01	-9.9 ± 7.6

OXFORD-LWT	58.1 ± 3.1	-11.9 ± 6.2	<0.01	-8.0 ± 8.6

^1 ^= ((calculated value from the corresponding prediction equation-reference metabolic parameter/reference metabolic parameter)*100)

^2 ^= Proximity to zero calculated as the difference between the reference metabolic parameter and the calculated value from the corresponding prediction equation (± 2 standard deviations) [13]

## Discussion

This is the first time were direct 24-hour energy expenditure measurements in healthy infants with a standardized methodology [6], was used as a reference to test the accuracy of several previously published prediction equations [1-4] for calculating 24-hour energy expenditure, resting and sleeping metabolic rates. In this comparison the weight based prediction equations for calculating 24-hour energy expenditure, resting and sleeping metabolic rates, derived from the short-duration metabolic measurements in the EMTAC, agreed with their respective reference values. Moreover, the length-weight based prediction equation for sleeping metabolic rate, derived from similar metabolic measurements in the EMTAC, also agreed with its respective reference value. However, neither of the length-weight based prediction equations for calculating resting and sleeping metabolic rate, as derived from short-duration metabolic measurements in the EMTAC, were in agreement with their respective reference values. Finally, the World Health Organization, Schofield or Oxford prediction equations for calculating resting metabolic rate were not in agreement with the respective reference values. Some of the problems encountered in the derivation of these early equations included data obtained from measurements utilizing closed circuit indirect calorimetry [3]. There were many problems associated with the closed circuit technique including the absorption of carbon dioxide not allowing for the calculation of the respiratory quotient [14], hyperventilation due to the subject knowledge of air being re-circulated and no direct measurement of oxygen. Furthermore, most of the laboratory technicians did not record whether the subject was post absorptive and/or in a relaxed state prior to resting metabolic rate measurements. Moreover, many of the early measurements of resting metabolic rate were not conducted in a thermo-neutral environment where the room temperature was kept between 22-27 degrees C [15]. Finally, a lot of the data were obtained in a limited number of ethnic groups. For example, much of the data utilized to derive the Schofield equations included a disproportionately large number Italians who have been found to have a higher resting metabolic rate per kg body weight [16]. As a result, the Schofield equations tended to over-estimate resting metabolic rate in many tropical ethnic groups by as much as 25% [5]. The minor ethnic group differences in body composition might also contribute to the World Health Organization [1] and Schofield [2] equations over estimating resting metabolic rate in many ethnic groups today [17].

In a previous study in our laboratory [4] we derived new prediction equations for calculating 24-hour energy expenditure, resting and sleeping metabolic rates in healthy infants utilizing the EMTAC instrument. Moreover, all metabolic measurements were conducted under standard conditions [4] at the same time in the morning between 9:00 AM and 1:00 PM. It is possible that variations in energy expenditure over the course of 24-hours, as shown by the presence of the metabolic circadian rhythm [6], might contribute to inherent inaccuracies when utilizing the World Health Organization [1], the Schofield [2] and the Oxford [3] prediction equations. Moreover, the metabolic measurement period of less than one hour might have also contributed to the inherent inaccuracies in these equations [1-3]. Despite using only the first four hours of metabolic data, the fact that measurements were conducted at the same time of day and were run at least three additional hours, as compared to the length of measurement when the World Health Organization [1], Schofield [2] and Oxford [3] prediction equations were derived, might have improved the consistency and accuracy of the metabolic data in the derivation of our new weight-based equations [4]. This is most likely due to the inclusion of some periods of increased physical activity and sleep in the infants [4]. This is further substantiated by the fact the infants have a two to four hour sleep-wake cycle between birth and six months of age [18].

These preliminary results suggest that the World Health Organization [1], the Schofield [2] and the Oxford [3] prediction equations may not be suitable for calculating caloric requirements in infants. Furthermore, both of the length-weight equations derived with the EMTAC instrument [4] for calculating energy expenditure and resting metabolic rate, respectively, were also not suitable for use in healthy infants. Moreover, these results suggest that additional 24-hour metabolic measurements need to be conducted in a greater number of infants from various ethnic groups. This will allow derivation of new equations that will be accurate for calculating energy requirements in healthy infants, accounting for all the metabolic variations that occur over a 24-hour period. Moreover, infants with various clinical disorders also need to be included such as those from our prior study in infants suffering from primary and secondary malnutrition [7].

In general both the length-weight prediction equations derived with the EMTAC instrument tended to over-estimate their respective metabolic parameters. This might be due to the fact that metabolic measures were performed in the morning [10,12], possibly representing the infants most active part of the day. This is further verified by the direct 24-hour metabolic measurements that showed a lower energy expenditure and physical activity during the evening and early morning hours [6]. However, the World Health Organization [1], the Schofield [2] and the Oxford [3] prediction equations greatly underestimated resting metabolic rate. The lack of standardized methods, limited number of subjects less than six-months old and some of the data being obtained over 80 years ago probably contributed to errors in their derivation and consistent under estimates in resting metabolic rate when utilized in today's infants.

## Conclusions

This is the first time actual 24-hour metabolic measurements in the Enhanced Metabolic Testing Activity Chamber (EMTAC) were used as a reference to evaluate several previously published prediction equations. We found those prediction equations by the World Health Organization, Schofield and Oxford, as well as the two length-weight based prediction equations from the EMTAC instrument, were inaccurate. However, the weight based prediction equations derived from our previous short-term metabolic measurements of four-hours in the EMTAC were accurate for calculating energy requirements in healthy infants up to six months of age.

## Abbreviations

BMI: Body Mass Index; EE: Energy expenditure; EMTAC: Enhanced metabolic testing activity chamber; F: Females; Kg: Kilograms; L/100: Conversion of L from meters to centimeters prior to calcuations; LWT: Length and weight; M: Males; OXFORD: Oxford; RMR: Resting metabolic rate; SCH: Schofield; SD: Standard Deviation; SMR: Sleeping metabolic rate; WHO: World Health Organization; WT: Weight; 24-h EE: Twenty-four hour extrapolated energy expenditure.

## Competing interests

The author declares that they have no competing interests.

## Authors' contributions

The author has contributed to the design and performance of the statistical analysis. Furthermore, the author participated in some of the previous studies that provided the data for this analysis. Moreover, the author also generated some of the grant proposals necessary for the financial support of this analysis and prepared the final version of this manuscript.

## References

[B1] World Health Organization (WHO) Energy and protein requirements, Report of a joint FAO/WHO/UNU Expert consultation 1985 Geneva: WHO Technical Report Series 724 3937340

[B2] Schofield WN Predicting basal metabolic rate, new standards and review of previous work Hum Nutr Clin Nutr 1985 39C 5S 41S 4044297

[B3] Henry CJ Basal metabolic rate studies in humans: measurement and development of new equations Public Health Nutr 2005 8 1133 1152 10.1079/PHN2005801 16277825

[B4] Duro D Rising R Cole C Cedillo M Valois S Lifshitz F New equations for calculating metabolic rate in infants J Ped 2002 140 534 539 10.1067/mpd.2002.123308 12032518

[B5] Henry CJK Rees DG New predictive equations for the estimation of basal metabolic rate in tropical peoples Eur J Clin Nutr 1991 45 177 185 1824042

[B6] Rising R Duro D Cedillo M Valois S Lifshitz F Daily metabolic rate in healthy infants J Ped 2003 143 180 185 10.1067/S0022-3476(03)00362-7 12970629

[B7] Rising R Sonmez GT Energy Expenditure and physical activity in recovering malnourished infants J Nutr & Met 2010 Article ID 171490 10.1155/2010/171490 PMC2911605 20700406

[B8] CDC Growth Charts National Center for Health Statistics. Health, United States, 2000 2000 Hyattsville, Maryland: Public Health Service

[B9] Jequier E Bjoerntorp P, Cairella M, Howard A Long-term measurement of energy expenditure in man: direct and indirect calorimetry In Recent advances in obesity research 1981 3 London: Libbey 130 135

[B10] Cole C Rising R Mehta R Hakim A Choudhury S Sundaresh M Lifshitz F Comprehensive assessment of the components of energy expenditure in infants using a new infant respiratory chamber J Am Coll Nutr 1999 18 233 241 10376779 10.1080/07315724.1999.10718857

[B11] Ravussin E Lillioja S Anderson TE Christin L Bogardus C Determinants of 24-hour Energy expenditure in man: Methods and results using a respiratory chamber J Clin Invest 1986 78 1568 1578 10.1172/JCI112749 3782471 PMC423919

[B12] Rising R Lifshitz F A lower metabolic rate in infants from obese biological mothers Nut J 2008 7 15 10.1186/1475-2891-7-15 PMC2423366 18485223

[B13] Bland JM Altman DG Statistical methods for assessing agreement between two Methods of clinical measurement Lancet 1986 327 307 310 10.1016/S0140-6736(86)91008-1 2868172

[B14] Durnin JVGA Basal Metabolic Rate in Man. Working paper submitted to the Joint FAO/WHO/UNU Expert Consultation on Energy and Protein Requirements 1981 Food and Agriculture Organization: Rome

[B15] Wilkerson JE Raven PB Horvath SM Critical temperature of unacclimatized male Caucasians J Appl Phy 1972 33 451 455 10.1152/jappl.1972.33.4.451 5075842

[B16] Hayter JE Henry CJK A re-examination of basal metabolic rate predictive equations: the importance of geographic origin of subjects in sample selection Eur J Clin Nutr 1994 48 702 707 7530655

[B17] Norgan NG Population differences in body composition in relation to the Body Mass Index Eur J Clin Nutr 1994 48 S10 S27 7843146

[B18] Balfour-Lynn IM Valman HB Practical Management of the Newborn 1993 London England: Blackwell Scientific Publications

